# Male involvement interventions influencing maternal reproductive health outcomes: a narrative synthesis using RE-AIM with implications for maternal mortality in Africa

**DOI:** 10.3389/frhs.2025.1659276

**Published:** 2025-10-23

**Authors:** Onyekachukwu Anikamadu, Ucheoma Nwaozuru, Nkiruka Obodoechina, Olufunto Olusanya, Temitope Ojo, Juliet Iwelunmor

**Affiliations:** ^1^ Department of Public Health Sciences, Brown School at Washington University in St. Louis, St. Louis, MO, United States; ^2^ Department of Implementation Science, Wake Forest University School of Medicine, Winston-Salem, NC, United States; ^3^ Division of Infectious Diseases, Washington University School of Medicine in St. Louis, St. Louis, MO, United States

**Keywords:** maternal reproductive health, interventions, male involvement, re-aim, Africa

## Abstract

**Introduction:**

Male involvement is crucial in optimizing maternal reproductive health outcomes, offering the potential to bolster reproductive health outcomes for mothers. The Reach, Effectiveness, Adoption, Implementation, and Maintenance (RE-AIM) framework can describe the implementation of interventions focused on promoting male involvement in maternal reproductive health. This study aims to (1) examine the implementation of male involvement interventions that influence maternal reproductive health outcomes and (2) report the implementation outcomes as conceptualized in the RE-AIM framework.

**Methods:**

This protocol followed the preferred reporting items for systematic review and meta-analysis. We searched PubMed, CINAHL, PsycINFO, and Web of Science utilizing a systematic review with narrative synthesis methodology to identify studies describing interventions that promote male involvement in maternal reproductive health outcomes in Africa from 2000 to 2024 Furthermore, we evaluated the public health impact of male involvement interventions from selected studies using the RE-AIM framework. Two reviewers independently screened articles, selected eligible studies, and extracted data. The quality of included studies was assessed using the NIH Assessment Tool for Observational Cohort and Cross-Sectional Studies.

**Results:**

This review included six studies that met the inclusion criteria. Overall, the studies reported increased maternal reproductive health indicators (e.g., antenatal care uptake, antiretroviral medication adherence, and postnatal care uptake) after implementing the male involvement-focused interventions. The most commonly reported RE-AIM dimensions were Reach (83.4%) and Efficacy/Effectiveness (70%). Adoption (40.5%), Implementation (38.9%), and Maintenance (13.3%) were less often reported. All studies reported on measures of primary outcomes, intervention duration and frequency, sample size, and participants’ characteristics. However, few reported on implementation fidelity, quality of life, methods used to identify staff, staff inclusion/exclusion criteria, implementation cost, and maintenance indicators.

**Conclusions:**

The review underscores the potential of male-involvement interventions in advancing maternal reproductive health outcomes. However, the limited reporting of external validity indicators such as intervention fidelity, intervention cost, and maintenance indicators limits such interventions’ scalability and long-term sustainability. This calls for more focus on reporting external validity indicators to inform the scalability and transferability of such interventions in real-world settings.

**Systematic Review Registration:**

https://www.crd.york.ac.uk/PROSPERO/view/CRD420251031192, PROSPERO CRD420251031192.

## Introduction

More than half a million women still die annually from pregnancy-related causes, with Sub-Saharan Africa (SSA) accounting for almost 50% of these deaths (1, 2). One of the key priorities of the United Nations (UN) Sustainable Development Goals (SDGs) target (3.1) is to reduce the global maternal mortality ratio (MMR) to fewer than 70 maternal deaths per 100,000 live births by 2030, with no individual country exceeding 140 deaths (3, 4). Prior research indicates that 73% of maternal deaths are due to direct obstetric causes such as hemorrhage, hypertensive disorders, and sepsis (3). Nearly 40%–45% of maternal deaths occur between the start of labor and the 24 h period immediately after birth (4, 5). Understanding factors influencing the timing of maternal death has significant importance in planning health programs and setting priorities.

To date, research focused on addressing maternal reproductive health outcomes has generally targeted women, showing significant variability in their effectiveness in reducing maternal mortality and related outcomes. To reach women and impact their maternal reproductive health outcomes, few studies have targeted men as agents of change, though the effectiveness of male involvement in interventions is unclear (6–8). This is because pregnancy and childbirth continue to be regarded exclusively as women's affairs in most African countries (1, 9). Prior research continues to highlight how men are absent during antenatal care and are often not expected in the labor room during delivery (1, 9–13). Yet, men in most African countries play a significant role in decision-making in domains of private life, particularly in women's choice for health-seeking behavior (14).

Men are also recognized as an integral part of the health system's response to delays in seeking care, reaching hospitals, and accessing appropriate care (15). Several studies have attempted to increase male involvement during maternal health care with the goal of decreasing the high burden of maternal mortality (6, 8). However, commonly identified barriers to male involvement include sociocultural norms, gendered roles, and lack of knowledge about reproductive and maternal health (15–17). In a cross-sectional community-based survey in Northwest Ethiopia, Mersha (2018) found that men's knowledge about obstetric danger signs and preparation for birth, preparedness, and complication readiness was poor (8). Of 824 men surveyed, only 42% were aware of obstetric danger signs, with 40.5% accompanying their spouses to antenatal care and a low percentage of 24.4% to the facility for delivery (8). Similarly, among 384 men surveyed in the Wakiso district of Uganda, only 6% of men accompanied their wives for antenatal checks (15). Several reasons were cited as barriers to male involvement in antenatal care, including men's busy schedules, the social culturalization of pregnancy and childbirth as women's responsibilities, and long waiting times (18).

This gap underscores the need for systematic evaluation of male involvement interventions in Africa. Despite men's well-documented role in decision-making, limited evidence exists on how interventions engaging men affect maternal reproductive health outcomes. Addressing this gap is critical for informing strategies that can reduce maternal mortality and strengthen the design and implementation of maternal health programs in resource-constrained settings. This review applies the RE-AIM framework to male involvement in maternal reproductive health outcomes to determine the extent of their involvement as agents of change with maternal mortality and related outcomes. While several implementation frameworks such as CFIR and TDF are commonly used to identify barriers and facilitators, we selected RE-AIM because it provides an evaluative structure that moves beyond determinants to capture internal and external validity (19). The RE-AIM framework includes indices of reach, effectiveness, adoption, implementation, and maintenance, which assist researchers with designing and evaluating interventions and external validity components (19). Reporting on external validity elements in interventions can help to understand: 1) delivery or whether interventions aimed at involving men were delivered as intended; 2) receipt or who received these interventions; and 3) enactment or whether intended recipients used the interventions (20, 21). Gaps at any of these stages of implementation could result in a difference in interventions for male involvement as intended and interventions received, which in turn may have implications for effectively addressing maternal mortality and related maternal reproductive health outcomes. Inadequate reporting of these elements may limit the generalizability of interventions across settings, thus hindering the translation of research to practice.

Additionally, because men matter and can serve as an intervention agent for reducing maternal mortality and improving overall maternal reproductive health outcomes, complete reporting using the RE-AIM components can provide insights most likely to be adopted by men and other key stakeholders in different African countries. This systematic review with narrative synthesis aims to (1) examine the implementation of male involvement interventions that influence maternal reproductive health outcomes and (2) evaluate these interventions on implementation outcomes as conceptualized in the RE-AIM (reach, efficacy/effectiveness, adoption, implementation, and maintenance) framework.

## Methods

This protocol will follow the preferred reporting items for systematic review and meta-analysis (PRISMA) (22). The PRISMA 2020 Checklist can be found in the Supplementary Files.

### Search strategy

We employed a systematic review methodology to systematically search four electronic databases: Medline/PubMed, PsycINFO, CINAHL, and Web of Science. We incorporated peer-reviewed articles published from January 2000 to August 2024 for papers that met the inclusion criteria. We selected the study period to capture over two decades of evolving global focus on male involvement in maternal and reproductive health care. We used a combination of controlled vocabulary and Boolean-paired keywords relating to male participation, maternal reproductive health care, maternal mortality, and interventions. Additionally, we reviewed the bibliographies of selected studies for other relevant citations.

### Inclusion and exclusion criteria

The search strategy was developed based on the PICO framework: Population (studies investigating maternal reproductive health interventions); Exposure (male involvement); Comparator (maternal reproductive health interventions without male involvement); Outcomes (maternal reproductive health outcomes e.g., miscarriage, spontaneous preterm birth, low birth weight, preterm premature rupture of membranes, pregnancy-induced hypertensive disorders and intrauterine growth restriction) (23). We included research studies that met the following criteria: 1) the paper discussed male involvement in maternal reproductive health outcomes in Africa; 2) the paper involved men with the goal of addressing maternal reproductive health outcomes or maternal mortality rates; 3) an intervention was evaluated; 4) paper was published in English. Exclusion criteria included: non-male involvement in maternal reproductive health, maternal mortality, or interventions in Africa; 2) conference abstracts, dissertations, editorials, and papers written in languages other than English. There was no limit to the publication date. We incorporated observational studies: cohort (retrospective and prospective), case-control, and cross-sectional studies.

### Study selection procedures

Two reviewers independently reviewed the titles and abstracts of non-duplicative studies to assess their eligibility for a full-text review. Thus, study titles or abstracts that did not meet the PICO criteria were excluded. Subsequently, they reviewed the full-text studies to determine if they met the eligibility criteria. The reviewers discussed disagreements regarding the articles screened and referred them to a third reviewer for dispute resolution.

### Risk of bias assessment

To assess the internal validity of the studies included in this review, we employed the Cochrane Collaboration's Risk of Bias assessment tool, which evaluates six key domains: selection bias, performance bias, detection bias, attrition bias, reporting bias, and other potential sources of bias (24, 25). Two reviewers (O.A. and U.N.) independently assessed each included study using the standardized criteria provided by the Cochrane Handbook. Each domain was rated as having a low, high, or unclear risk of bias. Discrepancies between reviewers were resolved through discussion to ensure consistency in judgment and to reach consensus. The risk of bias assessment was used solely to evaluate the methodological rigor and internal validity of the included studies; no study was excluded from the review based on its risk of bias score. A summary of the methodological assessment across the six studies is presented in Table 1.

**Table 1:** Methodological assessment table.

	Jones et al. 2021 (29)			Peltzer et al. 2020 (30)			Doyle et al. 2018 (31)			Daniele et al. 2018 (28)			Theuring et al. 2016 (32)			Nyondo et al. 2015 (27)		
Criteria	Yes	No	Other	Yes	No	Other	Yes	No	Other	Yes	No	Other	Yes	No	Other	Yes	No	Other
Was the study described as randomized, a randomized trial, a randomized clinical trial, or an RCT?	Yes			Yes			Yes			Yes			Yes			Yes		
Was the method of randomization adequate (i.e., use of randomly generated assignment)?	Yes			Yes			Yes			Yes				No		Yes		
Was the treatment allocation concealed (so that assignments could not be predicted)?	Yes			Yes			Yes			Yes				No		Yes		
Were study participants and providers blinded to treatment group assignment?			No			No			No			No			No			No
Were the people assessing the outcomes blinded to the participants’ group assignments?			Other			Other		No				Other		No				Other
Were the groups similar at baseline on important characteristics that could affect outcomes (e.g., demographics, risk factors, co-morbid conditions)?	Yes			Yes			Yes			Yes			Yes			Yes		
Was the overall drop-out rate from the study at endpoint 20% or lower of the number allocated to treatment?		No			No		Yes			Yes			Yes			Yes		
Was the differential drop-out rate (between treatment groups) at endpoint 15 percentage points or lower?		No			No		Yes			Yes			Yes			Yes		
Was there high adherence to the intervention protocols for each treatment group?	Yes			Yes			Yes			Yes			Yes			Yes		
Were other interventions avoided or similar in the groups (e.g., similar background treatments)?	Yes			Yes			Yes			Yes			Yes			Yes		
Were outcomes assessed using valid and reliable measures, implemented consistently across all study participants?	Yes			Yes			Yes			Yes			Yes			Yes		
Did the authors report that the sample size was sufficiently large to be able to detect a difference in the main outcome between groups with at least 80% power?	Yes			Yes			Yes			Yes			Yes			Yes		
Were outcomes reported or subgroups analyzed prespecified (i.e., identified before analyses were conducted)?	Yes			Yes			Yes			Yes			Yes			Yes		
Were all randomized participants analyzed in the group to which they were originally assigned, i.e., did they use an intention-to-treat analysis?	Yes			Yes			Yes			Yes			Yes			Yes		

### Data extraction and management

Data were extracted from eligible studies into an electronic spreadsheet following the eligibility assessment of all full-text articles. The following data were extracted from selected studies meeting eligibility criteria: study characteristics (author, sample, study design, comparison/control components, intervention components, assessment, outcome variable, and outcomes). We used a narrative synthesis to describe the studies meeting the eligibility criteria. We define narrative synthesis here as an approach to synthesizing findings from multiple sources using words and texts from the sources to summarize and explain the sources (26). Prior research suggests that using narrative synthesis in cases of statistical meta-analysis or another specialist form of synthesis (such as meta-ethnography for qualitative studies) is not feasible, particularly due to substantial methodological and clinical heterogeneity between studies identified (26). Furthermore, we extracted the reach, effectiveness, adoption, implementation, and maintenance indicators using the RE-AIM framework as a guide.

For this study, Reach is defined as the extent to which the male involvement intervention engaged its target population, including participant characteristics and recruitment strategies. Effectiveness is the impact the intervention had on maternal reproductive health outcomes. Adoption is the extent to which participants, communities, or organizations (e.g., health centers) adopt the male involvement intervention. Implementation assesses the consistency and fidelity with which the intervention was delivered, while Maintenance evaluates whether outcomes were sustained over time at individual and organizational levels.

## Results

We narratively synthesized the included studies, summarized, and discussed the findings of the included studies.

### Inclusion and exclusion of studies

**Figure 1:**
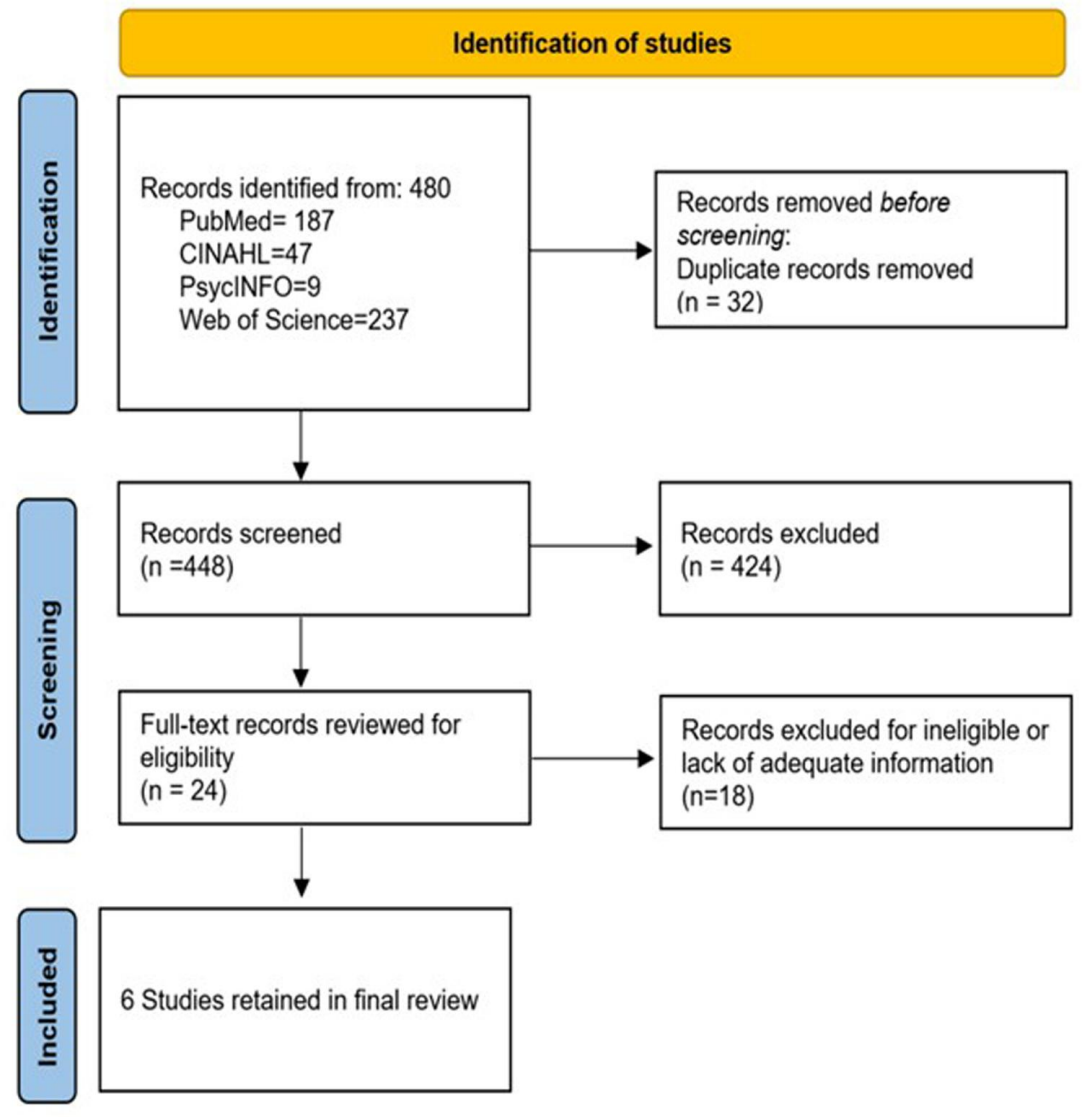
Flow chart diagram of search results.

The electronic database searches retrieved 480 records (187 from PubMed, 47 from CINAHL, nine from PsycInfo, and 237 from Web of Science). After eliminating the duplicates in EndNote (*n* = 32), 448 studies remained for title and abstract screening. During this initial screening phase, 424 studies were excluded as they did not meet the inclusion criteria based on their titles and abstracts. They mainly were general HIV studies examining HIV status testing, general knowledge and attitudes, and treatment adherence. Twenty-four records were selected at the abstract level to undergo full-text review to assess their eligibility for inclusion in the review. During this phase, 18 studies were excluded as they did not meet the predefined eligibility criteria upon full-text evaluation. Ultimately, six studies/interventions were deemed eligible and included in the systematic review on male involvement in maternal reproductive health outcomes. During the full-text review, qualitative studies that solely focused on individual experiences, perceptions, or attitudes of males/partners/spouses related to maternal reproductive health were removed, and cross-sectional studies were excluded if they could not capture the dynamic and longitudinal aspects of male involvement. Figure 1 provides a flowchart of the study selection process.

### Study characteristics

The final sample consisted of six studies published between 2015 and 2024, all of which evaluated interventions designed to improve male involvement in maternal and reproductive health outcomes. The sample size for these studies ranged from 462 (27) to 1,144 (28). Two of the studies were located in South Africa (29, 30), one in Malawi (27), one in Burkina Faso (28), one in Rwanda (31), and one in Tanzania (32). Table 2 provides details on the description of the studies included in the review.

**Table 2:** Summary of study characteristics.

First Author/Year	Country/Region	Study design	Study duration	Study size	Participants’ age	Participants’ employment	Participants’ level of education	Intervention characteristics	Male involvement indicators	Results	Recommendations
Jones et al. 2021 (29)	Mpumalanga Province, South Africa	Random cluster trial	2014–2017	Total 1,399	Phase 1: 18+ women with HIV Phase 2: 18+ women with HIV and male partner	Unemployed 76.6% Employed 23.4%	0–9 Grade 20.5% 10–11 Grade 48.1% 12 Grade +31.4%		Attendance at antenatal care visits. Engagement in preventing mother-to-child HIV transmission. Support for mothers during post-partum.	Male partner involvement improved drug adherence. Male partner support can contribute to better maternal outcomes.	More research is needed between depression and poor maternal adherence and suboptimal provision of medication to infants. More research so needed between maternal and infant adherence behaviors.
Peltzer et al 2020 (30)	South Africa	Randomized controlled trial	April 10–January 30, 2017	1,370 (Total) 696 (control) 714 (intervention)	Average age of 28+ women	Unemployed 76.8%	Up to the 9th grade, 20.7% Up to the 10 to 11 grade 48.2% Up to the 12 grade and beyond 31.1%		Attendance at health visits Engagement in PMTCT educational sessions. Emotional and financial support. Support for mothers during post-partum.	High levels of depressive symptoms among HIV-infected perinatal women. Male partner participation contributed to additional benefits towards the reduction of depressive symptoms.	Further research on the perinatal period is needed to clarify what constitutes meaningful or impactful male involvement.
Doyle et al. 2018 (31)	Rwanda (4 districts) Karongi Musanze—Nyaruguru Rwamagana	Randomized controlled trial	February 19–March 17, 2015	1,199 (Total) 624 (control) 571 (intervention)	21–35 men	Self-employed 89.29% Employed/ earning a wage 9.96% Out of work 0.75%	None 9.37% Some primary 53.47% Primary complete 23.18% Secondary or higher 13.97%		Increased attendance at antenatal care visits. Increased open discussions about pregnancy, childbirth, and postnatal care Reduction in intimate partner violence.	Women in the intervention group reported attending slightly more antenatal visits. More men participated in antenatal visits. More women reported higher use of modern contraception and higher levels of partner support during pregnancy. Reduced maternal stress and violence, which are critical factors for maternal mental health.	Culturally adapted male engagement interventions can be effective at changing gender inequalities and a range of health-related behavioral outcomes.
Daniele et al. 2018 (28)	Bukina Faso (5 districts) Bolomakote Guimbi Ouezzinville Secteur	Randomized controlled trial	February 16–June 12, 2015	1,144 (Total) 561 (control) 583 (intervention)	15–45 women	No work outside the home 38.3% Street vendor 43.1% Craftswoman 7.5% Shopkeeper 6.9% Other 4.1%	No education 51.5% Primary school 27.4% Above primary school 21.1%		Male partners attended educational and counseling sessions. Increased support of antenatal and postnatal care. Involvement in decision-making relating to maternal health issues.	Increased postnatal care attendance Increased exclusive breastfeeding among the participants. Improved care-seeking behavior.	The study recommends expanding male involvement in maternal health care by addressing socio-cultural barriers and integrating similar interventions into the healthcare system.
Theuring et al. 2016 (32)	Mbeya, Tanzania	Randomized controlled trial	April and May 2013	199 (Total) 102 (control) 97 (intervention)	18–44 women	Employed 74.7% Unemployed 25.3%	Up to Primary 65.7% At least secondary 34.3%		Increase male partner attendance at antenatal care visits. 23.59% of male partners came back with their partners after one visit. Male partner participation decision decision-making of women's health care during pregnancy.	Women in the intervention group reported increased involvement of male partners during their pregnancy, including during antenatal care visits. Women also experience improved support from their partners during pregnancy.	Invitation cards passed through pregnant women can increase male participation in treatment services. Invitation cards could be an effective strategy that could be implemented into policy to increase uptake of male participation in implementation studies.
Nyondo et al. 2015 (27)	Blantyre, Malawi	Randomized controlled trial	June 14–December 2013	462 (Total) 232 (control) 230 (intervention)	16+ women	Not employed 85.9%	Some education 77.9% -No education 22.1%		Increase male partner attendance at antenatal care visits. 28.26% of women were accompanied by their male partner in the intervention group. Male partner participation in HIV testing and counseling.	Increased involvement of male partners. Improved engagement in PMTCT services. Increased rates of HIV testing and counseling can improve maternal health outcomes	The use of invitation cards and addressing cultural and healthcare barriers can help sustain male participation in maternal and child health care.

### Intervention and theoretical framework

These studies demonstrate a diverse array of interventions, reflecting a comprehensive effort to address the complex dynamics of how male engagement was incorporated within maternal health interventions. Only one of the studies explicitly included a theoretically informed intervention to improve male involvement in maternal reproductive health outcomes (31). Doyle and colleagues employed sociological theories of gender and masculinity that highlight how gender inequalities are reproduced–or transformed–through “everyday interactions in [the] home (31). The intervention used a structured 15-session curriculum adapted from Program P, an open-source manual for engaging men in maternal and child health, created by Promundo, CulturaSalud, and REDMAS, which includes a curriculum for fathers/couples, community resources for designing health provider training, and community campaigns. Daniele and colleagues utilized different charts for counseling sessions adapted from existing counseling tools produced by the World Health Organization and the Ministry of Health of Senegal (28). Nyondo et al. (27) utilized invitation cards guided by the results of their study's formative phase, which used the PRECEDE-PROCEDE model, a planning model that reinforces a participatory approach, to identify strategies of male involvement (27).

### Study design

Five of the six studies used randomized controlled trials (RCTs) to evaluate the impact of male partner involvement on maternal and child health outcomes (27–31). Three used individual-level RCTs (27, 28, 31) while two applied cluster RCTs (29, 30). The remaining study employed a controlled quasi-experimental intervention trial at the facility level without random assignment (32).

### Interventions implemented

In Daniele et al (28), the intervention encompassed three distinct components to enhance male partner involvement in the maternal care period. The first component involved interactive group discussion sessions exclusive to male partners (28). These discussions were facilitated by health workers and revolved around narratives of fictional couples facing challenges due to a lack of communication and health information. These sessions, conducted in French and local languages, stimulated dialogue and understanding among three to 13 participants. The second component comprised individual couple counseling sessions during pregnancy, which took place in private consultation rooms with one or two health workers. These sessions covered various topics related to pregnancy, childbirth, and postpartum care. Discussions included the significance of antenatal and postnatal care, birth preparedness, danger signs for mothers and newborns, exclusive breastfeeding, family planning, and contraception. Interactive tools, including a flip chart with illustrations and relevant texts, facilitated effective communication between health workers and couples. The third component was postnatal couple counseling sessions occurring approximately 6 h after childbirth if the delivery occurred at a primary healthcare center. These sessions offered an opportunity for couples to further discuss relevant information pertaining to the postpartum period. Importantly, discussions also centered on contraception, enabling couples to make informed decisions regarding family planning. The same flip chart from the initial counseling session was utilized for continuity.

In a multi-site randomized controlled trial conducted in Rwanda by Doyle and colleagues (31), the Bandebereho couples’ intervention was assessed for its impact on male engagement in reproductive and maternal health. Over 21 months, 1199 men from local communities participated, undergoing structured questionnaires and follow-up surveys at 9 and 21 months. The intervention implemented by Rwanda Men's Resource Center (RWAMREC) aimed to transform masculinity norms through 15 sessions for men and their partners, focusing on topics like gender dynamics, fatherhood, communication, caregiving, and male engagement in health. The study's rigorous design, data collection method, and extensive follow-up contributed to a comprehensive understanding of the intervention's potential to reshape gender dynamics and promote positive models of fatherhood for improved maternal and child health outcomes. Jones et al. (29) sought to evaluate the impact of male involvement on enhancing the prevention of mother-to-child transmission outcomes (29). The research employed a two-phase, two-condition experimental or control cluster randomized controlled trial period. Only women were engaged during phase one, whereas phase two included female and male partners. Baseline assessments occurred between 6 and 30 weeks of pregnancy, followed by participation in antenatal group intervention sessions led by lay healthcare workers. Postpartum, two individual sessions were conducted. Antenatal reassessments were conducted at 32 weeks of pregnancy, and post-natal evaluation took place at six weeks, six months, and 12 months.

The randomized controlled trial by Nyondo and colleagues (27) conducted in Blantyre, Malawi, aimed to assess the feasibility and effectiveness of using invitation cards to enhance male partner involvement (MI) and prevention of mother-to-child transmission (PMTCT) of HIV services among pregnant women attending antenatal care period the study included two groups: Group A utilized invitation cards for MI in PMTCT. At the same time, Group B employed word-of-mouth invites. Pregnant women up to 30 weeks of gestation were enrolled from South Lunzu and Mpemba health centers, with the primary outcome being the proportion of women attending PMTCT services with their partners. The invitation card, developed based on formative research and literature, was seen as a more plausible strategy for MI by study participants.

Peltzer et al. (30), conducted a longitudinal clinic cluster randomized control trial investigating the impact of male involvement on PMTCT uptake and depressive symptoms among perinatal rural women living with HIV in South Africa. The intervention implementation employed a 2-phase, two-condition design. In this intervention, pregnant women living with HIV with male partners were enrolled in phase one, while women only were enrolled in phase two, including both partners. The Protect Your Family (PYF) intervention was led by lay health care workers and comprised tender Pacific antenatal group sessions, individual or couple sessions, and postpartum sessions. Audio Computer-Assisted Self-Interview Software (ACASI) assessments were conducted, and a control group received time-matched video presentations. Spanning recruitment from April 10th, 2014, to January 30th, 2017, the study aimed to understand the influence of male involvement on PMTCT and maternal wellbeing, contributing to insights into the potential benefits of engaging male partners in PMTCT services within the rural South African context.

In a multi-site implementation study on partner involvement in Mbeya Region, Tanzania, a controlled intervention trial was conducted at Ruanda Health Centre to assess the effectiveness of invitation letters for male partner engagement (32). The study involved pregnant women attending their first antenatal care (ANC) visit between April and May 2013, with criteria including confirmed pregnancy and accessible partners. Participants were assigned to intervention or control groups using a quasi-randomized approach. Interviews conducted in Swahili covered socio-demographics, and HIV status knowledge was self-reported. The intervention group received a written invitation letter for partners, while the control group was instructed to verbally invite their partners to the next ANC session. A joint ANC session was offered if partners attended, and couple voluntary counseling and testing sessions were provided (CVCT). Follow-up interventions explored partner attendance and reasons for non-attendance. The study aimed to assess the impact of invitation letters on male partner involvement in ANC sessions and CVCT uptake, contributing insights into strategies for enhancing male engagement in maternal health services.

### Male involvement measurement

Male involvement was measured differently across the studies. In the study conducted by Daniele et al. (28), male engagements were based on the attendance and participation of male partners in the three educational sessions provided as part of the intervention. “Good relationship adjustment” 8 months postpartum was also measured using the Dyadic Adjustment Scale (33) and the Locke-Wallace Marital Adjustment Test (34), which determined a woman's satisfaction with her partner and the degree of communication, shared decision-making, and agreement between the couple on issues related to reproductive health. Doyle et al. (31) measured men's involvement by participating in and assessing the impact of the Bandebereho couples’ intervention. Jones et al. (29) and Peltzer et al. (30) adapted a Male Involvement Index (35), assessing male participation during pregnancy. Nyondo and colleagues (27) assessed male involvement by measuring the proportion of pregnant women accompanied by their partners at weeks 2 and 6 of the study after receiving an invitation card. Theuring et al. (32) measured partner involvement by partner return rate and CVCT rate.

### Summary of study findings

The findings of all interventions were relatively similar across all studies. Daniele et al. (30) reported that the intervention led to a follow-up rate exceeding 96%, with 74% of couples attending at least two study sessions. Moreover, attendance at postnatal care consultations was notably higher in the intervention group than in the control group (CI: 6.0–17.5) and involving men as supportive partners correlated with improved adherence to recommended postpartum practices. Doyle et al. (31) reported substantial improvements attributed to the Bandebereho intervention, which showed greater male accompaniment at antenatal care (IRR 1.50, *p* < 0.001), and heightened participation of men in childcare and household tasks. In Jones et al. (29) study, Mail involvement was associated with self-reported maternal or infant antiretroviral therapy (ART) adherence, highlighting its relevance during pregnancy in the antenatal clinic setting.

Additionally, findings supported using male involvement and depression treatment as supplementary strategies to enhance maternal and infant medication uptake as part of the PMTCT protocol. Nyondo et al. (27) revealed that the invitation card group exhibited a 50% higher likelihood of being accompanied by male partners to antenatal care clinics compared to the standard of care (SoC) group (RR: 1.49; 95% CI: 1.06–2.09; *p* = 0.02). Peltzer et al. (30) found that interventions combining multi-session PMTCT and male partner participation reduced depressive symptoms among perinatal HIV-positive women. Theuring et al.'s (32) study involving invitation letters resulted in 30.9% of male partners returning for antenatal care, indicating the efficacy of these simple measures in increasing male partner attendance.

### RE-AIM indicators

The individual interventions encompassed 9–14 (with a median of 14) out of the 27 RE-AIM indicators. None of the examined interventions encompassed all 27 indicators across the five dimensions of the RE-AIM framework. The proportions of reporting varied across dimensions, with the highest average reporting rates observed for reach (83.4%), followed by efficacy/effectiveness (70%), adoption (40.5%), implementation (38.9%), and the lowest reporting rates were for maintenance (13.3%). Table 3 summarizes the overall percentage of studies reporting on each dimension of the RE-AIM framework, while Table 4 presents specific implementation outcomes from each of the selected studies as conceptualized within the RE-AIM framework.

**Table 3:** The proportion of male involvement intervention studies in maternal reproductive health outcomes reporting reach, efficacy/effectiveness, adoption, implementation, and maintenance (RE-AIM) indicators and components.

RE-AIM dimensions and components	Reporting frequency (*N* = 6)	Reporting proportion (*N* = 6)
Reach		
• Method to identify the target population	5	83.3%
• Inclusion criteria	6	100.0%
• Exclusion criteria	4	66.7%
• Sample size	6	100.0%
• Participation rate	4	66.7%
• Characteristics of participants	6	100.0%
• Characteristics of non-participants	4	66.7%
*Average of overall reach dimensions*	*5.0*	*83*.*34%*
Efficacy/Effectiveness		
• Measures of the primary outcome for at least one follow-up	6	100.0%
• Intent to treat utilized	4	66.7%
• Quality-of-life measure	0	0.0%
• Baseline activity reported	6	100.0%
• Percent short-term participant attrition	5	83.3%
*Average of overall efficacy/effectiveness dimensions*	*4.2*	*70*.*0%*
Adoption		
• Description of intervention location	6	100.0%
• Description of staff delivering the intervention	5	83.3%
• Method to identify staff	0	0.0%
• Level of expertise of delivery staff	4	66.7%
• Inclusion criteria/exclusion criteria for setting and staff	0	0
• Adoption rate (setting level)	0	0.0%
• Adoption rate (participant level)	2	33.3%
*Average of overall adoption dimensions*	*2.43*	*40*.*5%*
Implementation		
• Intervention duration and frequency	6	100.0%
• The extent to which protocol was delivered as intended	0	0
• Measures of cost of delivery	1	16.7%
• *Average implementation dimensions*	*2.33*	*38*.*9%*
Maintenance		
*Individual level-maintenance*		
• Was individual behavior assessed ≥6 months post-intervention	4	66.7%
• Measures of long-term attrition	0	0.0%
*Program level-maintenance*		0.0%
• Current status of the program [If and how the intervention was adapted long-term (which elements were retained after the program was completed)]	0	0.0%
• Some measure/discussion of alignment with organization/setting	0	0.0%
• Cost of maintenance	0	
*Average of overall maintenance dimensions*	*0.80*	*13*.*3%*

**Table 4:** Implementation outcomes from selected studies as conceptualized in the RE-AIM framework.

First Author/Year	Reach	Effectiveness	Adoption	Implementation	Maintenance
Jones et al. 2021 (29)	1,399 HIV positive pregnant women who had male partners in two health districts in the Mpumalanga province.	Improving drug adherence (ART) of pregnant HIV women with participation of their male partners.	Lay community health workers were used to carry out the implementation in antenatal clinics. Similar antenatal clinics could adopt interventions to increase male involvement.	Women between 6 and 30 weeks of pregnancy were invited for four group intervention sessions (control sessions) antenatally and two post-partum individual sessions. Women were re-assessed antenatally at 32 weeks of pregnancy and postnatally at 6 weeks, 6 and 12 months * Phase 1, only women participated, while in phase 2, both women along their male partners	More research on male involvement in drug adherence treatments is encouraged. Sustainability of this intervention depends on cultural barriers
Peltzer et al. 2020 (30)	1,370 HIV infected pregnant women from 12 communities in rural South Africa.	The study looks into depressive symptoms during the pregnancy and postnatal period of participants. The study findings showed 40% of the women had depressive symptoms prenatally and 30% postnatally.	Lay community health workers were used to carry out the implementation. The intervention faced stigma and intimate partner violence, which can impact the adoption of this intervention in similar settings.	Participants attended gender-specific antenatal group sessions led by lay healthcare workers, followed by one antenatal individual or couple session and two postpartum individual or couple sessions * Phase 1, only women participated, while Phase 2, both women along their male partners	HIV stigma and intimate partner violence remain a challenge. Increasing Male involvement during pregnancy will help sustain the intervention.
Doyle et al. 2018 (31)	1,199 expectant and/or fathers of children under 5 years old.	Male participation iin antenatal visits and provided more support during pregnancy. After the intervention, couples had better communication skills, understanding of gender roles, and improved healthcare engagement.	Community volunteers and community health workers- sex matched interviewers with no involvement in the intervention conducted the interviews. The adoption rate by healthcare providers was high, indicating the potential for the adoption of the intervention in similar settings.	Structured questionnaires were administered to male participants. Follow-up surveys were conducted with men and their current partners at 9 months and again at 21 months. The male participants attended 15 sessions, while their partners attended 8 sessions	Improved involvement of male partners and a decrease in intimate partner violence.
Daniele et al. 2018 (28)	1,144 women from 3 health centers, cohabiting with male partners.	Increase of male partner participation in postnatal care by 11.7%. There were also increased rates of exclusive breastfeeding.	Participation rates of the male partners during the three sessions were high, showing strong adoption rates for the intervention group.	The intervention included group and couple counseling sessions.	Follow-up visits were conducted at 3 and 8 months postpartum, and breastfeeding and care-seeking rates remained stable.
Theuring et al. 2016 (32)	199 pregnant women and their male partners.	Written and verbal invitation to antenatal care sessions, effectiveness of male partner involvement during and after a pregnancy.	Research assistants used strategies to increase male involvement during and after pregnancy, showing potential for widespread adoption within healthcare settings.	Pregnant women were given interviews after routine antenatal care visits. Male partners were invited to antenatal care visits verbally or with invitation cards. Verbal invitations were slightly more feasible, while written invitations were used for follow-up instruments for non-attenders.	Intervention maintenance is low cost and can be sustained over time.
Nyondo et al. 2015 (27)	462 pregnant women who attended antenatal care without their male partners.	Invitation cards significantly increased male involvement in antenatal care.	Research assistants used the invitation card strategy to implement the intervention.	Pregnant women were given invitation cards to give to their partners, inviting them to attend antenatal care treatment.	Sustaining the intervention in the long term could be effective in increasing male involvement in PMTCT services

#### Reach

The average proportion reporting across indicators within the reach dimension was 83.4%. Within the reach dimension, study participants’ inclusion criteria 6 (100%), sample size 6 (100%), and participants’ characteristics 6 (100%) were reported in all the interventions included in this review. Five of the six studies (83%) reported methods used to identify the target population, including recruitment in healthcare clinics and facilities (27–29, 31, 32). The included studies had a variety of inclusion criteria for study participants, including eligibility based on: age (28–31), being pregnant (27–30, 32), relationship status, requiring being in a relationship or cohabiting (27–32), gestational age (27, 28),, and being HIV positive (29, 32). Exclusion criteria of participants were reported by four of the six studies (66.7%). Exclusion criteria ranged from being a widow or divorced, not living with HIV, age, and gestational age outside the study limits. Sample size, defined as the number of participants who completed the study, ranged from 199 (32) to 3,500 (29).

Participation rates, determined by the number of participants recruited who participated in the intervention, were reported by four (66.7%) of the studies. Characteristics of nonparticipants include being out of work (31), miscarriage/ infant death and other birth complications (29, 32), relocation/ changing health care facilities (29, 32), and transportation challenges to health facilities (30, 32).

#### Efficacy/effectiveness

On average, efficacy/effectiveness indicators were reported at 70% across all six interventions (27–32). Of the five indicators of this dimension, measures/results of the primary outcome for at least one follow-up and baseline activity reported were the most recorded indicators of study 6 (100%), followed by percent short-term participation attrition 5 (83.3%), utilization of intent to treat analysis 4 (66.7%), and quality of life measure as the least 0 (0%).

Regarding outcome measures, five of the six studies included male involvement (27, 29–31), and return rate (32), as primary outcome measures for male involvement, whereas Daniele and colleagues (28), measured relationship adjustment as a secondary measure. Jones et al. (29) and Peltzer et al. (30) used an adapted male Involvement Index to assess male participation during pregnancy. Baseline activities were reported for all studies (100%), which included interview sessions, and structured questionnaires, which helped determine male participation at baseline and capture demographic and socioeconomic characteristics such as age, parity, ethnicity, religion, occupation, and educational level, on their reproductive health history and male partner's characteristics. Intent-to-treat analysis was utilized in 4 (66.7%) interventions to assess intervention uptake and follow-up (27, 28, 31, 32). The Attrition rate was reported by 5 of the studies (83.3%) with results in alignment of nonparticipants. None 0 (0%) of the interventions reported on having a measure for the quality of life among study participants.

#### Adoption

The average reporting proportion of adoption indicators across the studies was 40.5%. The description of the intervention location was the most reported adoption indicator, with a report rate of 100%. Five intervention locations were in health care centers and facilities (27–30, 32), while one was in a school administrative office (31). Five (83.3%) of the studies reported the description of staff who implemented the intervention. Staff ranged from midwives, fathers who served as community volunteers, lay workers, and trained research assistants. Of the five studies, only 4 stated some staff training, but none (0%) explicitly stated their staff level of expertise. Staff responsibilities included delivering parts of the intervention, educating participants, and facilitating intervention sessions.

Two (33.3%) studies reported the adoption rate on the participant level (27, 31). Doyle et al. (31) stated that the proportion of participants who adopted the recommended behaviors increased between 6.4 and 11.7 percentage points for each of the three primary outcomes and between 4.8 and 8.7 percentage points for secondary outcomes. Nyondo et al. (27), expected to observe an increase in MI in PMTCT services from 2% (without intervention) to 12% (with intervention). No study 0 (0%) reported on methods to identify staff, inclusion/exclusion criteria for the setting and staff, and adoption rate at the setting level.

#### Implementation

Implementation was one of the least reported dimensions of the RE-AIM framework (38.9%). The most commonly reported indicator was the study's duration and frequency, while also reporting the intervention's format, 6 (100%) (27–32). Doyle et al. (31), was the only study of the six to report on measures of cost delivery. The study mentioned that participants received a 2000 Rwandan franc transport stipend (approximately $2.50 USD) for each interview. No study 0 (0%) reported the extent to which their protocol was delivered as intended.

#### Maintenance

The least reported dimension was maintenance, split into two indicators, individual-level maintenance and program-level maintenance, with a combined reporting average of 13.3%. Individual-level behavior assessment more than or equal to 6 months post-intervention was the sole sub-indicator reported in the individual-level maintenance indicator 4 (66.7%). Follow-ups ranged from 8 months to 21 months post-intervention. The level of long-term attrition was not reported 0 (0%). No studies reported on indicators concerning program-level maintenance 0 (0%).

### Quality of the selected studies assessed

The methodological quality of the six selected studies was assessed using a standardized evaluation tool covering key aspects of study design, implementation, and analysis. All six studies were described as randomized or controlled trials, with five explicitly using randomized designs (27–31), and one applying a quasi-randomized approach (32). Randomization procedures were adequately described in five studies, and allocation concealment was implemented in four (27–29, 31). None of the studies blinded participants or providers to group assessments due to the nature of the interventions, and only one study (28) indicated probable blinding of outcome assessors, though not with certainty. The lack of blinding across most studies introduces potential performance and detection bias. These limitations reduce confidence in subjective outcomes which we therefore interpret results for self-reported outcomes more cautiously.

Most studies demonstrated strong adherence to intervention protocols, utilized valid and reliable outcome measures, and implemented intention-to-treat analysis. Sample size calculations with at least 80% power were reported in five of the six studies (27–31), while all studies identified outcomes prior to analysis. Four studies (27, 28, 31, 32) reported overall and differential dropout rates within acceptable limits, while Jones (29) and Pelzer (30) experienced higher attrition, particularly in Phase 1. Despite some limitations, the overall methodological rigor of the included studies was moderate to high, with most studies addressing core elements of trial validity and reliability. A summary of findings can be found in Table 4.

## Discussion

This review analyzed and synthesized empirical evidence on male involvement in maternal reproductive health outcomes. The findings from the six intervention studies in the review provide evidence of the value of male participation in improving maternal reproductive health outcomes in Africa. The few intervention studies also highlight the limited focus on interventions geared toward promoting male involvement in enhancing maternal productive health outcomes. Therefore, there is a need to expand the evidence base on the role of male-involvement interventions on maternal reproductive health outcomes. In addition, the review of the six intervention studies included in the review emphasized the need to report internal validity dimensions of RE-AIM (i.e., reach and effectiveness) and low reporting of external validity dimensions of RE-AIM (i.e., adoption, implementation, and maintenance). Additionally, the limited reporting of fidelity, cost, and maintenance likely reflects persistent systemic challenges in African contexts. Recent evidence from Malawi shows that program fidelity and sustainability are jeopardized by high implementation costs, supply shortages, labor constraints, and adverse conditions (36). This finding is consonant with other review studies that have found the under-reporting of external validity dimensions of RE-AIM (26–39). To advance beyond the focus on the effectiveness of an intervention, a clear and comprehensive reporting of all aspects of intervention implementation is essential to enhance the scalability and translatability of such interventions in other settings and groups (19). In addition, several methodological limitations affect how these findings should be interpreted. The absence of blinding in most of the included studies may overestimate effects on self-reported behaviors, while high or differential attrition reduces confidence in some outcomes. For this reason, outcomes that were objectively measured can be considered more reliable than those based solely on self-report.

The review also highlights the benefits of male involvement in improving maternal reproductive health outcomes. The studies included in this review reported enhanced maternal health outcomes, such as increased postnatal care attendance (28), maternal antiretroviral therapy (ART) adherence, and (29) overall well-being of women with intentional male involvement in the maternal reproductive health process. This is consistent with other studies that have reported improved maternal reproductive health outcomes, such as higher rates of ANC and PNC attendance (40, 41) with male involvement in maternal health. While these studies were cross-sectional evaluations, they support the findings of the intervention studies in this review.

In the studies included in the review, strategies such as education and training on how to support the women were provided to men to better equip them to provide support to enhance maternal reproductive health outcomes, addressing the challenge of awareness, frequently cited as a barrier to male involvement in women's health (42–44). This indicates that for men to be better equipped to be part of the solution for maternal reproductive health outcomes, there is a need to enhance their self-efficacy through awareness, training, guidance, and support (16, 45). Besides improving knowledge of the importance of male involvement in maternal reproductive health, interventions should include strategies that address cultural and structural barriers to male involvement (45). These comprehensive measures are essential in overcoming systemic and cultural barriers that impede male involvement in maternal reproductive health outcomes (16, 42, 44). Consequently, beyond improving maternal reproductive health outcomes, increased male involvement may potentially enhance men's health, particularly for men with limited contact with formal health systems. This could be an entry point for preventative services for men.

Additionally, the review shows variability in the level of male involvement, underscoring the need for further exploration of the meaning and levels of male involvement needed for optimal maternal reproductive health outcomes (29). This is imperative to strike a balance between male involvement and women's autonomy in seeking reproductive health services. While male engagement can improve maternal reproductive health outcomes, it also risks reinforcing unequal gender power relations if not carefully designed. Gender-transformative approaches that promote equitable decision-making and safeguard women's autonomy are essential to ensure interventions do not inadvertently undermine women's agency (31). Understanding and implementing strategies that maximize the benefits of male involvement in maternal reproductive health outcomes while minimizing potential drawbacks, such as compromising women's autonomy in health decision-making, is critical (41, 46). Considering a broader perspective, it would be worth considering moving beyond an instrumentalist approach to men's involvement in maternal reproductive health and taking a gender-transformative approach that takes into account the complex interplay of social, cultural, biological, environmental, political, and economic determinants of women's health (31, 47–49). Such an approach, as supported by existing literature (47–49), could lead to a more comprehensive and holistic understanding of improving maternal reproductive health outcomes that maximizes women's assets, resources, and social support, including men.

Furthermore, regarding implementing the interventions to promote male involvement in maternal reproductive health outcomes documented in this review, there was variability in reporting the RE-AIM indicators. Most of the included studies reported on reach (83%) and intervention effectiveness (70%), some reported on intervention adoption (41%), while a few reported on intervention implementation (39%) and maintenance (13%). Regarding the *reach* dimension, the documentation of indicators such as participation rate, characteristics of participants and non-participants, and methods to identify the target population provides insights into the accessibility and acceptability of an intervention. In the review, the study participants were mainly recruited from health facilities and community centers. The characteristics of participants, inclusion criteria, and sample size were reported in all the studies, while indicators such as non-participation and characteristics of non-participants were infrequently reported. The poor reporting of indicators of this RE-AIM dimension has implications regarding the internal and external validity of male involvement interventions for maternal reproductive health outcomes and raises concerns regarding the generalizability of the results and understanding of who such interventions might be most suitable for. Additionally, it is also possible that publication bias influenced our findings. Interventions with null or negative results may be less likely to be published, which could lead to an overrepresentation of studies reporting positive effects. Given the small pool of studies included, this bias may further limit the generalizability of our conclusions.

The second most frequently reported dimension within the RE-AIM framework was effectiveness. Effectiveness indicators related to outcome measures and the effect of the intervention on primary or secondary outcomes (e.g., measures/results of the primary outcome for at least one follow-up and baseline activity reported) were reported in all the studies. All the studies reported increased promotion of maternal reproductive health outcomes post-intervention implementation. However, none of the studies reported on quality of life measures, a trend consistent with other studies that have reported low or no reporting of this indicator (37, 38). This observation warrants consideration regarding the precise definition of this indicator or the need for clarification regarding its measurement. This step is crucial to better understand its relevance as a potential indicator for assessing reach.

In contrast to the relatively high reporting of effectiveness, the indicators related to the adoption and implementation of the intervention were sparsely reported in studies, which poses a challenge in translating findings to larger populations and diverse settings (50). Details on intervention adoption are needed to gauge an intervention's suitability and appropriateness. Most included studies reported on intervention location and staff characteristics (e.g., description of staff credentials and level of expertise), only two reported on the adoption rate at the participant level, and no studies reported on the adoption rate at the setting level.

Regarding implementation, all the studies reported on the intervention duration and frequency. However, none of the studies reported on the intervention fidelity. Omitting information about the intervention fidelity limits the external validity of the interventions. Furthermore, only one of the studies explicitly stated the cost incurred during the implementation of the intervention, which included transportation costs to the intervention location (31). Cost is a critical aspect of interventions designed for and implemented in low-resource areas. Reporting on the implementation cost is essential to understand how resources were utilized (37). This offers insights into the potential sustainability of the intervention. Also, maintenance indicators were largely missing due to a lack of reporting on the institutionalization of the programs.

This present narrative synthesis has several strengths. To our knowledge, it is one of the first to provide a narrative synthesis on the implementation of interventions focused on promoting male involvement in maternal reproductive health outcomes. This review quantitatively estimates external and internal validity reporting across the interventions. We also utilized a comprehensive search strategy for this review. However, there are some limitations to our current review. Our review of the study is limited to the information reported in the publication. Some studies may have collected but not reported the analyzed indications. To minimize this bias, we reviewed all companion articles (51) focused on the included interventions and examined them for potential data.

## Conclusion

In conclusion, our findings show that male involvement in intervention studies demonstrated some effect on increasing adherence to ART medication and antenatal and postnatal visits among women. This underscores the potential of male-involvement interventions in advancing maternal reproductive health outcomes. However, the limited reporting of external validity indicators such as intervention fidelity, intervention cost, and maintenance indicators limits such interventions’ scalability and long-term sustainability. This calls for more focus on reporting external validity indicators to inform and support the scalability and transferability of such interventions in real-world settings. Future implications include the need for stakeholders to embed more implementation outcome assessments into intervention design and reporting. Doing so, can strengthen external validity and support development of male involvement interventions that are not only effective but also scalable, sustainable, and equitable.

## Data Availability

The original contributions presented in the study are included in the article/Supplementary Material, further inquiries can be directed to the corresponding author.
